# Compliance to spectacle use in children with refractive errors- a systematic review and meta-analysis

**DOI:** 10.1186/s12886-020-01345-9

**Published:** 2020-02-24

**Authors:** Nonita Dhirar, Sankalp Dudeja, Mona Duggal, Parul Chawla Gupta, Nishant Jaiswal, Meenu Singh, Jagat Ram

**Affiliations:** 1 Department of Community Medicine, PGIMER, Chandigarh, India; 2 Department of Pediatrics, PGIMER, Chandigarh, India; 3 Department of Community Ophthalmology, Advanced Eye Centre, PGIMER, Chandigarh, India; 4 Advanced Eye Centre, PGIMER, Chandigarh, India

**Keywords:** Compliance, Spectacles, Refractive error, Children

## Abstract

**Background:**

Primary objective of this review was to measure compliance with spectacle use in children with refractive errors. Secondary objective was to understand the reasons for non-compliance.

**Methods:**

The databases searched were Ovid, EMBASE, CINAHL and Pubmed. All studies up to March, 2018 were included. The search terms were- ((((((Compliance [Title/Abstract]) OR Adherence [Title/Abstract]) OR Compliant [Title/Abstract]) OR Adherent [Title/Abstract])) AND (((Spectacle [Title/Abstract]) OR Spectacles [Title/Abstract]) OR Eye Glasses [Title/Abstract])) AND ((((Child [Title/Abstract]) OR Children [Title/Abstract]) OR Adolescent [Title/Abstract]) OR Adolescents [Title/Abstract]). Two researchers independently searched the databases and initial screening obtained 33 articles. The PRISMA guidelines were followed for conducting and writing the systematic review. Two reviewers assessed data quality independently using the Quality Assessment tool for systematic reviews of observational studies (QATSO). Poor quality studies were those, which had a score of less than 33% on the QATSO tool. Sensitivity analysis was done to determine if poor quality studies effected compliance. Galbraith plot was used to investigate statistical heterogeneity amongst studies. A random effects model was used to pool compliance.

**Results:**

Twenty-three studies were included in the review, of which 20 were included in the quantitative analysis. All the studies were cross sectional. The overall compliance with spectacle use was 40.14% (95% CI- 32.78-47.50). The compliance varied from 9.84% (95% CI = 2.36–17.31) to 78.57% (95% CI = 68.96–88.18). The compliance derived in sensitivity analysis was 40.09%. Reasons for non-compliance were broken/lost spectacles, forgetfulness, and parental disapproval.

**Conclusion:**

Appropriate remedial measures such as health education and strengthening vision care services will be required to address poor compliance with spectacle use among children.

## Background

Uncorrected refractive errors are a major cause of morbidity globally. Recent data shows that uncorrected refractive error is among the leading causes of moderate or severe vision impairment in the global population in 2015 [1].It is estimated that in developing countries, 7 to 31% of childhood blindness and visual impairment is avoidable [2]. In the age group of 5–15 years, nearly12.8 million (0.97% global prevalence) children are visually impaired due to uncorrected or unsatisfactorily corrected refractive errors [3].Theprojected cost of uncorrected refractive error (RE) described as direct and indirect loss of world productivity is 269 billion international dollars (I$) (US$ 202 billion), and the projected cost of addressing the issue is US$ 28 billion over 5 years [4, 5].Children suffering fromrefractive errors viz., myopia, amblyogenic hyperopia, astigmatism and anisometropia require appropriate treatment at the earliest [5, 6].Uncorrected refractive errors in children lead to poor academic growth, injuries, reduced social participation, and functional impairment [7].Correction of visual impairment with spectacles is the most cost-effective intervention for improving eye care and thus the productivity and functionality of children. Spectacles have a quality of being simple to use, non-invasive and inexpensive. However, the benefit of these visual aids depends on the compliance by end users.

A number of studies are available worldwide to look into the factors determining compliance with spectacle use [8–27]. Studies have shown that the compliance with spectacle use in children with visual impairment due to REs is only one third or less [11, 16, 17, 19, 24]. Compliance remained low even when the spectacles were provided for free, and poorer rates were observed in older children [14, 18, 21, 23, 24]and children residing in rural areas [13, 15]. Poor follow up after school-based screenings, broken spectacles, loss, forgetfulness [9, 11, 13–15, 17–20, 23, 24, 27] parental and children’s perceptions [8, 11, 13, 15, 16, 18, 22–24],peer pressure [9, 11, 14, 18, 19, 22, 24], safety concerns and the patient’s self-esteem are few of the reasons cited for poor compliance.

Variable rates of compliance worldwide suggest that augmented attention is warranted, including investment in development and assessment of spectacle compliance interventions to assist in reducing complications associated with non-wear of spectacles. Based on literature review, we hypothesized that compliance with spectacle use would be low in children. The primary objective of the present review is to study the compliance with spectacle use in children with REs and to arrive at a summary measure of the rate of compliance by pooling data from various studies. The second objective was to assess the reasons for non-compliance in children with RE.

## Methods

This systematic review was conducted in accordance with the Meta- analysis of Observational Studies in Epidemiology [28] (MOOSE) guidelines and the Preferred Reporting Items for Systematic Reviews and Meta-analyses standard (PRISMA) [ 29].

### Literature search

An extensive search was conducted on published literature and efforts were made to acquire information about the unpublished literature from conference proceedings, unpublished research and from topic experts. The searches were performed on28^th^ May, 2017 and were updated on 31st March, 2018 on the following databases: Ovid, EMBASE, CINAHL and Pubmed. A thorough search of title and abstract (tiab) using the key words was performed. We did MeSH terms screening along with use of Boolean operators. The search terms were-((((((Compliance [Title/Abstract]) OR Adherence [Title/Abstract]) OR Compliant [Title/Abstract]) OR Adherent [Title/Abstract])) AND (((Spectacle [Title/Abstract]) OR Spectacles [Title/Abstract]) OR Eye Glasses [Title/Abstract])) AND ((((Child [Title/Abstract]) OR Children [Title/Abstract]) OR Adolescent [Title/Abstract]) OR Adolescents [Title/Abstract]). Reference lists of articles retrieved in the initial step were screened for pertinent studies. Efforts were made to contact authors for articles, which could not be obtained. We also searched additional platforms like Google search for non-indexed studies.

### Inclusion criteria

Observational and experimental study designs were included in the systematic review. These included cross sectional, case control and cohort study designs. Experimental study designs were also explored. Studies assessing compliance with spectacle use in children with REs, published in English language, with one or more of the key words in the title or abstract were included in the review. Compliance was defined as regular use of glasses prescribed for refractive errors including myopia, hypermetropia and astigmatism, assessed either by observation or by interviewing the children. Studies irrelevant to the objective of this review e.g. in children suffering from other eye disorders, conducted in adults, not published in English language were excluded from the review. The studies in which raw data were missing or unclear were excluded from quantitative analysis. We did not include conference proceedings. The participants comprised of children of both sexes with REs.

### Data extraction

The studies were independently reviewed by two researchers (N.D, S.D), performed a thorough search of the databases and screened titles and abstracts based on the research question, and population and outcome in terms of compliance with spectacle use.. Compliance was defined as regular use of glasses prescribed for refractive errors including myopia, hypermetropia and astigmatism, assessed either by observation or by interviewing the children. Based on the initial screening, full-text articles were obtained. Duplicates were removed in the initial stages. The third investigator (M.D) solved any disagreement in the selection of studies. Two reviewers conducted quality assessment independently using the QATSO tool used in previous studies [30–32]. No significant difference was found in individual assessments of the reviewers. This study has been registered with PROSPERO, with registration number CRD42017068190.

### Data analysis

The data were entered separately from the included studies in a pre-designed and piloted format that recorded the information about author name, country of study, date of publication, study design, number of children, duration of follow up, type of study, percentage compliance and reasons for non-compliance.

After extraction, all related data were entered into Microsoft Excel for compilation. The data were analyzed with STATA MP 12 v11 [33]. Pooled compliance estimate for spectacle use in children was generated using a forest plot. Compliance rates were calculated from raw proportions or percentages reported in the selected studies. The raw proportions/percentages were pooled using a random-effects model and pooled estimates and the 95% Confidence intervals (CI) were calculated. Correlation coefficient was calculated between per capita GDP expenditure and percentage expenditure on health with percentage compliance. In addition compliance was measured on basis of setting of the study i.e., whether the children were provided spectacles in screening settings in the field or in clinical setting. Sensitivity analysis was done to assess the effect of poor-quality studies on the overall compliance with spectacle use. Galbraith plot was used to investigate the statistical heterogeneity amongst the studies.

## Results

### Study characteristics

**Fig. 1:**
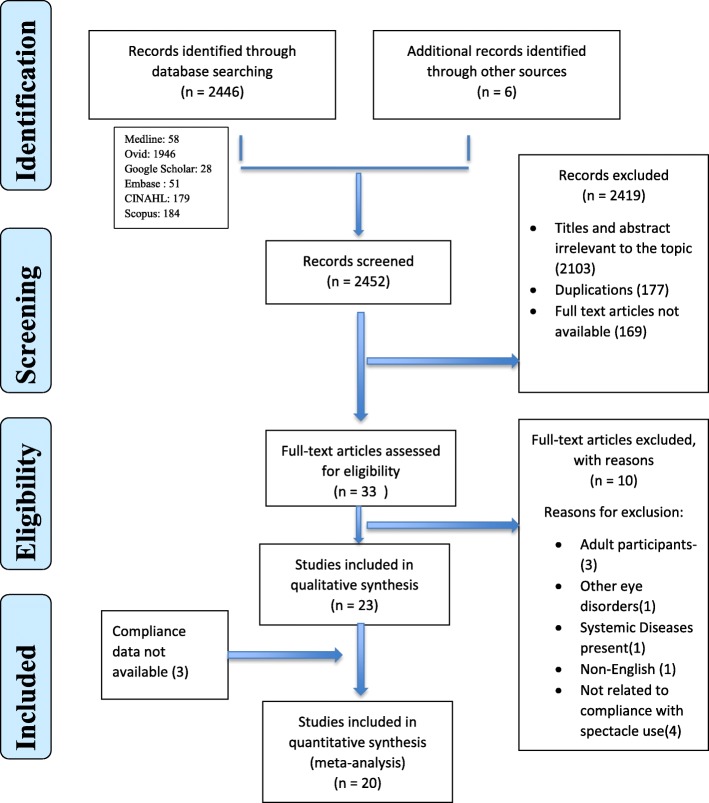
Flow of selection of studies for the review

A total of 2452articles were retrieved, of which 33 articles met the study criteria for inclusion (Fig. 1). After construing the full text, we finally included 23 studies for the review (Table 1). We did not include data for pooled compliance estimate from three studies [8, 26, 34] as the proportion of children compliant with spectacle use was not available. However, descriptive data from these studies was included.

**Table 1:** Characteristics of selected studies included in systematic review and meta-analysis (*N* = 23)

S. No	Authors	Country	Age	Year	Study Design	WHO Region^a^	World Bank Income^b^	Per capita GDP (2017 in $)	HDI	Expenditure on Health (% of GDP)	Compliance Measure	Type of prescription	Duration of Follow-up	Sample size	No. Compliant (%)
1.	Narayanan A et al	India	13–17 years	2017	Cross Sectional (Qualitative)	SEAR	LMIC	1940	0.640	3.89	Questionnaire/ Interview based	No specific type		32	
2.	Bhatt NK et al	India	6–15 years	2017	Cross Sectional	SEAR	LMIC	1940	0.640	3.89	Wearing spectacles on surprise visit	No specific type	3 months	200	78 (39.0)
3.	Anwar I et al	Pakistan	11–16 years	2017	Cross Sectional	EMR	LMIC	1548	0.562	2.69	Questionnaire/ Interview based	Myopia, hypermetropia, astigmatism and compound	8–12 months	440	180 (40.9)
4.	Kumar MR et al	India	6–17 years	2017	Cross Sectional	SEAR	LMIC	1940	0.640	3.89	Wearing spectacles on surprise visit	Children with visual acuity < 6/9	6 months	82	52 (63.4)
5.	Bhandari G et al	Nepal	5–16 years	2016	Cross Sectional	SEAR	LIC	835	0.574	6.15	Wearing spectacles on surprise visit or in bag	No specific type	No follow up	170	48 (28.2)
6.	Sumana M et al	India	9–16 years	2015	Cross sectional	SEAR	LMIC	1940	0.640	3.89	Wearing spectacles on surprise visit	No specific type	6 months	362	139 (38.4)
7.	Al Shamarti S et al	Iraq	Pre-school	2015	Cross Sectional	EMR	UMIC	5166	0.685	3.40	Questionnaire /Interview based	No specific type	Not mentioned	114	41 (35.9)
8.	Von-Bischhoffs-hausen FB et al	Chile	4–19 years	2014	Cross Sectional	AMR	HIC	15,346	0.843	8.07	Wearing spectacles on surprise visit	Visual Acuity 20/40 in either eye, myopia 0.75 diopters (D), 1.5D of astigmatism, or hyperopia.	12 months	204	121 (59.3)
9.	Pavithra MB et al	India	7–15 years	2014	Cross Sectional	SEAR	LMIC	1940	0.640	3.89	Wearing spectacles on surprise visit	Children with myopia ≥ −0.5 spherical equivalent diopters in one or both eyes, hypermetropia ≥ + 1.00 spherical equivalent diopters in one or both eyes and astigmatism ≥1.00 D	3 months	83	48 (57.8)
10.	Gogate P et al	India	8–16 years	2014	Cross Sectional	SEAR	LMIC	1940	0.640	3.89	Wearing spectacles on surprise visit followed by interview	myopia ≥ −0.50D or hyperopia ≥ + 1.00D	6–12 months	1018	300 (29.4)
11.	Aldebasi YH et al	Saudi Arabia	7–13 years	2013	Cross Sectional	EMR	HIC	20,761	0.853	5.83	Wearing spectacles on surprise visit or in bag	A child with myopia of more than −0.50 Dioptre (D) or hypermetropia of more than + 1.00D are prescribed spectacles	6 months	631	209 (33.1)
12.	Megbelayin EO et al	Nigeria	9–21 years	2013	Cross Sectional (mixed methods)	AFR	LMIC	1969	0.532	3.56	Wearing spectacles on surprise visit	VA < 6/9 in at least one eye	No follow up	61	6 (9.8)
13.	Turcin LA et al	Romania	6–11 years	2013	Cross Sectional	EUR	UMIC	10,814	0.811	4.95	Wearing spectacles on surprise visit or in bag	Myopia (≥ − 1.00 DS in one or both eyes) Hyperopia (≥ + 1.50 DS in one or both eyes) Astigmatism (values ≥ 1.00 D)	12 months	315	82 (26)
14.	Messer DH et al	USA	8–14 years	2012	Cross Sectional (mixed methods)	AMR	HIC	59,532	0.924	16.84	Wearing spectacles on surprise visit	Myopia ≥ −0.75 D, astigmatism ≥1.00 DC, and anisometropia ≥1 D and hyperopia ≥ + 2.50 D	3 months	247	82 (33.1)
15.	Keay L et al	China	12–15 years	2010	Cross Sectional	WPR	UMIC	8827	0.752	5.32	Wearing spectacles on surprise visit or in bag	No specific type	1 month	415	204 (49.1)
16.	Li L et al	China	14–18 years	2010	Cross sectional (Qualitative)	WPR	UMIC	8827	0.752	5.32	Questionnaire/interview based	No specific type		28	
17.	Khandekar R et al	India	School going	2008	Cross Sectional	SEAR	LMIC	1940	0.640	3.89	Questionnaire/ Interview based	Myopia (> 0.75 D spherical unilateral or bilateral)	3–4 months	70	55 (78.5)
18.	Congdon N et al	China	11.4–17.1 years	2008	Cross Sectional	WPR	UMIC	8827	0.752	5.32	Questionnaire/ Interview based	Uncorrected VA of 6/12 or worse and a 25% random sample of subjects with VA better than 6/12	No follow up	1892	713 (37.7)
19.	Li L et al	China	Average 14.7 years	2008	Cross Sectional	WPR	UMIC	8827	0.752	5.32	Questionnaire/ Interview based	All children with presenting VA 6/12 in either eye and children with spectacles improving vision to 6/12	3 months	210	55 (26.2)
20.	Odedra N et al	Tanzania	Average 14 years	2008	Cross Sectional (mixed methods)	AFR	LIC	936	0.538	6.12	Wearing spectacles on surprise visit	VA worse than 6/12 in either eye or hyperopia of 2 diopters (D) or more	3 months	108	40 (37.0)
21.	Holguin AM et al	Mexico	6–18 years	2006	Cross Sectional	AMR	UMIC	8903	0.774	5.86	Wearing spectacles on surprise visit	Children with visual acuity 6/12 or less	4–18 months	493	66 (13.4)
22.	Khandekar R et al	Oman	6–17 years	2002	Cross Sectional	EMR	HIC	15,668	0.821	3.83	Wearing spectacles on surprise visit	No specific group	12 months	571	418 (73.2)
23.	Horwood AM et al	UK	1–7 years	1998	Cross Sectional	EUR	HIC	39,720	0.922	9.88	Questionnaire/ interview based	No specific group	Not mentioned	113	

^a^*SEAR* South East Asia Region; *EMR* Eastern Mediterranean Region; *WPR* Western pacific region; *AFR* African region; *AMR* Region of the Americas

^b^*UMIC* Upper middle income country; *LMIC* lower middle income country; *LIC* Lower income country; *HIC*: High income country

Empty cells depict missing information

*VA* Visual acuity

All the 23 studies included were cross-sectional in nature. The studies are geographically diverse and covered 14 countries. Most (34.7%) of the studies were from South-East Asian region [8, 10–12, 15, 19, 23],followed by Eastern Mediterranean region [9, 13, 18, 25] (17.4%). More number (39.1%) of studies was from lower-middle income country (LMIC) groups. The per capita GDP of the countries ranged from 835 USD for Nepal to 59,532 USD for USA. Percentage expenditure on health ranged from 2.69 for Pakistan to 16.84 for USA. In most studies, compliance with spectacles was defined if the child was wearing spectacles at the time of surprise visit by the investigators or had the spectacles in bag (65.2%). For a few studies compliance was defined by taking interviews of the children and asking questions regarding their patterns of spectacle use (34.8%). The prescription patterns varied across studies. Some studies used the cutoff of ≥ 0.5 Diopters for defining myopia and assessing compliance while a few defined myopia with a cut off of ≥ 1 Diopters. Similarly, for hyperopia the cut offs ranged from + 1.0 D to 2.5 Diopters. A few studies defined the prescriptions and cutoffs in terms of visual acuity and included children with visual acuity < 6/9 to < 6/12 across studies.

### Population characteristics

The total number of children studied was 7859. The children enrolled were from all age groups ranging from preschool to end of school. The period of follow up ranged from ‘no follow up’ to follow up of 18 months. The variation in ages was vast and contributed to significant heterogeneity.

### Quality assessment

The studies were assessed for the methodological quality based on the tool developed by Wong WC et al. [31, 32] Close to half (43.5%) of the studies were of good quality while 9 studies (39.1%) were of satisfactory quality and 4 studies (17.4%) were poor quality studies with score < 30%. The tool used for quality assessment is as shown in eTable 1 (supplementary file). The results of quality assessment of studies are attached as a supplementary file (eTable 2).

### Compliance with spectacle use

**Fig. 2:**
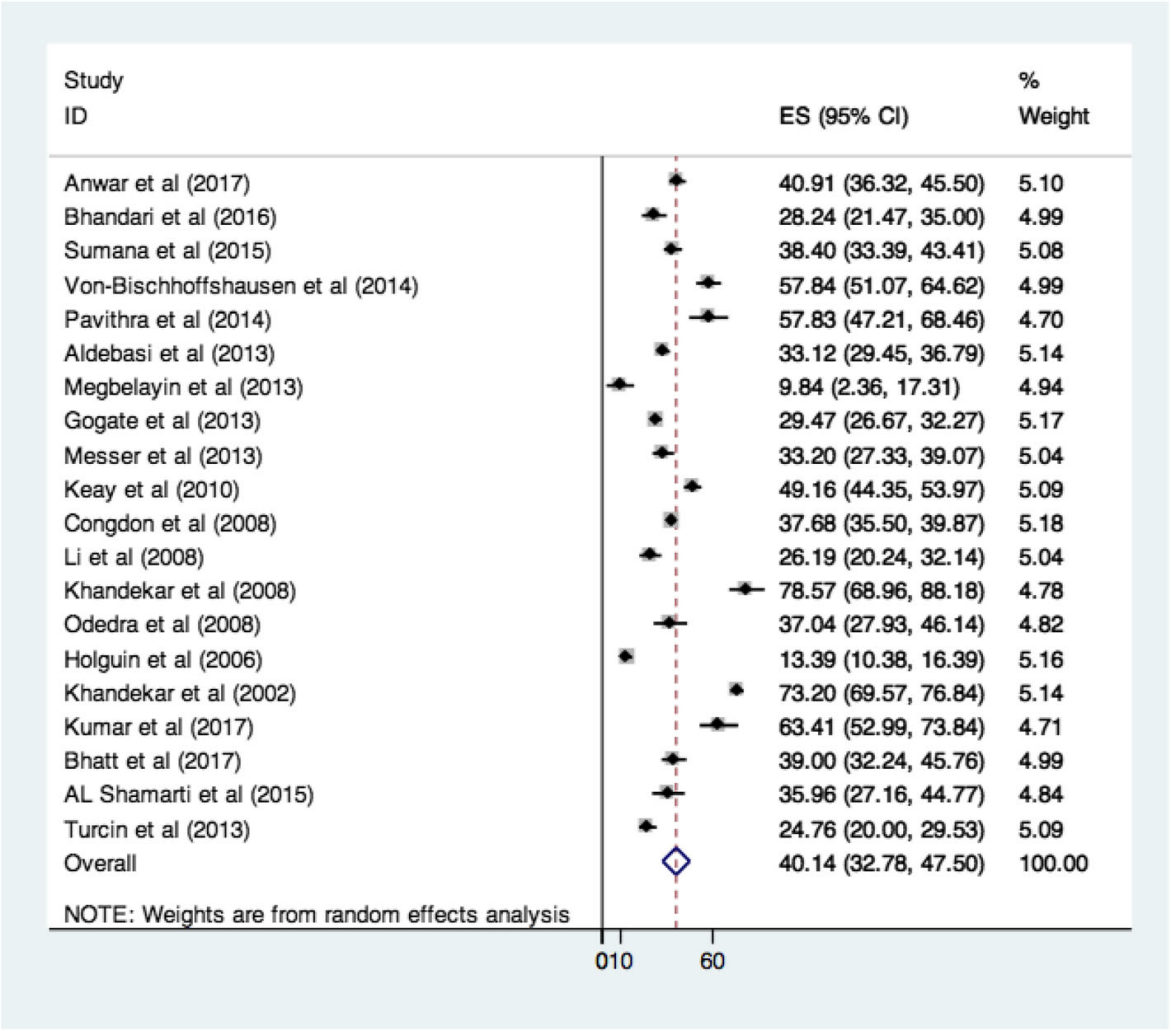
Compliance to Spectacle use (%) as reported by selected studies (*N* = 20)

The overall compliance with spectacle use was 40.14% (95% CI- 32.78-47.50) for 20 studies. The compliance varied from 9.84%(95% CI = 2.36–17.31) to 78.57%(95% CI = 68.96–88.18). Four studies had extreme values and their confidence intervals did not overlap those of other studies. Two of these studies reported very low compliance [16, 24] while 2 reported very high compliance 23, 25] with spectacle use. A forest plot of the studies and the compliance rate is as shown in Fig. 2. The compliance across almost all income groups was poor. The correlation coefficient for relationship between per capita GDP and percentage compliance was − 0.051, indicating a decrease in compliance with an increase in per capita income. However, this was not statistically significant (*p* = 0.830). Similarly, the correlation coefficient for relation between percentage expenditure on health and percentage compliance was − 0.238, indicating that an increase in percentage expenditure on health was associated with a decrease in compliance. This relationship also, was not statistically significant (*p* = 0.312). The prescription cut offs used for measuring compliance had an effect on the compliance pattern across studies. Based on the setting of the study the compliance was pooled for screening vs clinical care. It was observed that the compliance was 45.84% in the setting of clinical care and 39.33 in the setting of screening (eFig.1). This difference was not statistically significant. The method of measurement of compliance also varied across groups. A few studies used observation/ surprise visit as a measurement method while others used interviews/questionnaires for measurement of compliance. We grouped and analyzed the studies by compliance measure. The pooled compliance where observation was the method of measurement was 39.24% while the pooled compliance where interview was the method of measurement of compliance; the pooled compliance was 43.23% (eFig.2). This difference was not statistically significant, indicating that the measurement method did not significantly alter the compliance. A few studies that used a higher prescription cut off, reported better compliance as compared to studies that had lower cut offs for measurement of compliance, indicating lesser compliance at lower refractive errors. However most studies did not report any specific cut offs for prescriptions and some studies took cut offs using visual acuity, hence the pooled effect could not be observed.

### Sensitivity analysis and investigation for heterogeneity

**Fig. 3:**
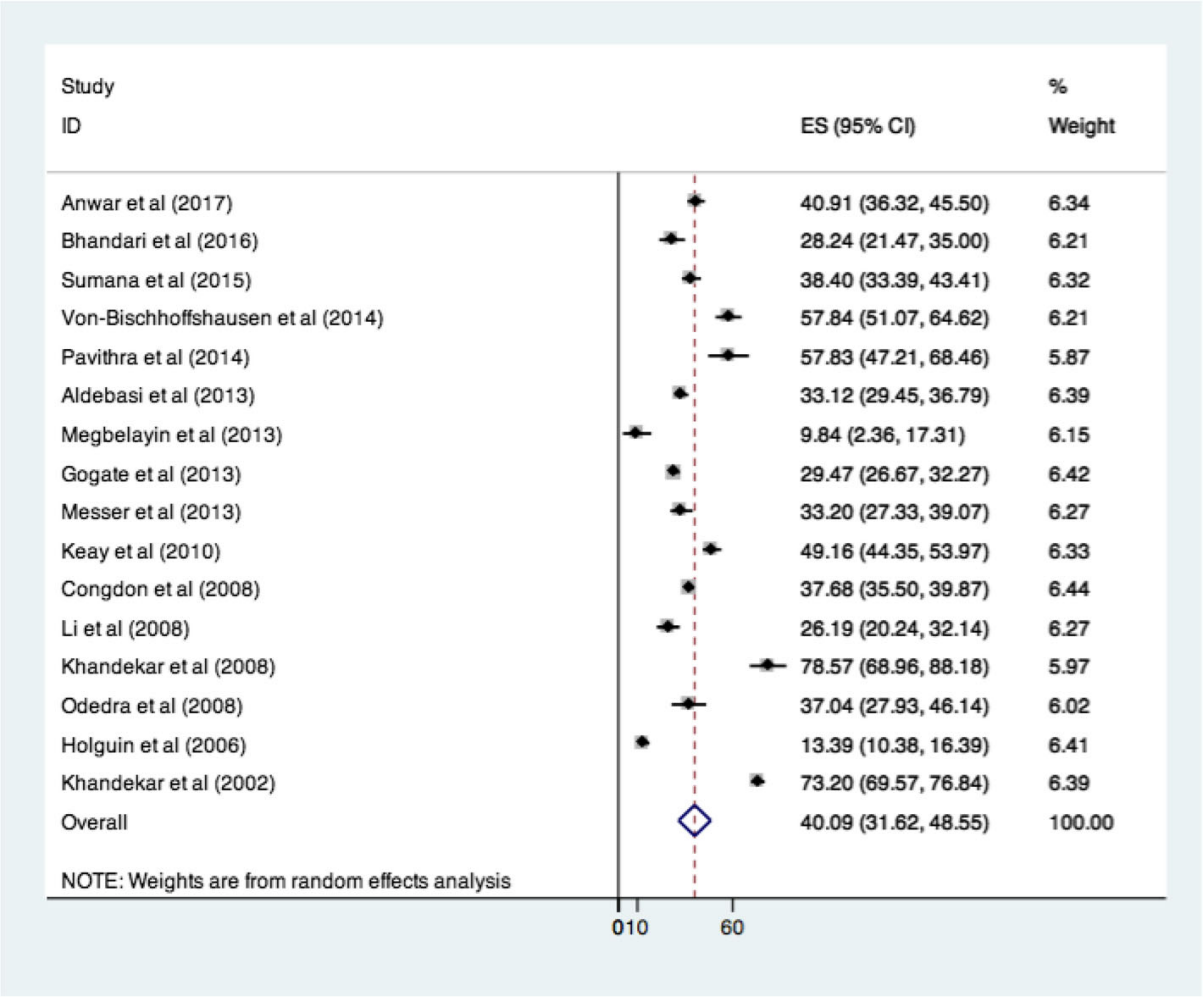
Sensitivity analysis showing compliance to spectacle use (%) after removal of poor quality studies (*N* = 16)

Sensitivity analysis was done to know whether the poor quality studies [10, 12, 13, 17] had any effect on the overall compliance. The overall compliance with spectacle use did not vary significantly and was 40.09% when these studies were removed from the analysis. The forest plot excluding these studies is as shown in Fig. 3. The Galbraith plot (eFig.3) has shown that the studies had significant heterogeneity. The heterogeneity could be attributed to varying sample sizes and prescription cut offs (degree of refractive errors).

### Reasons for non-compliance with spectacle use

**Fig. 4:**
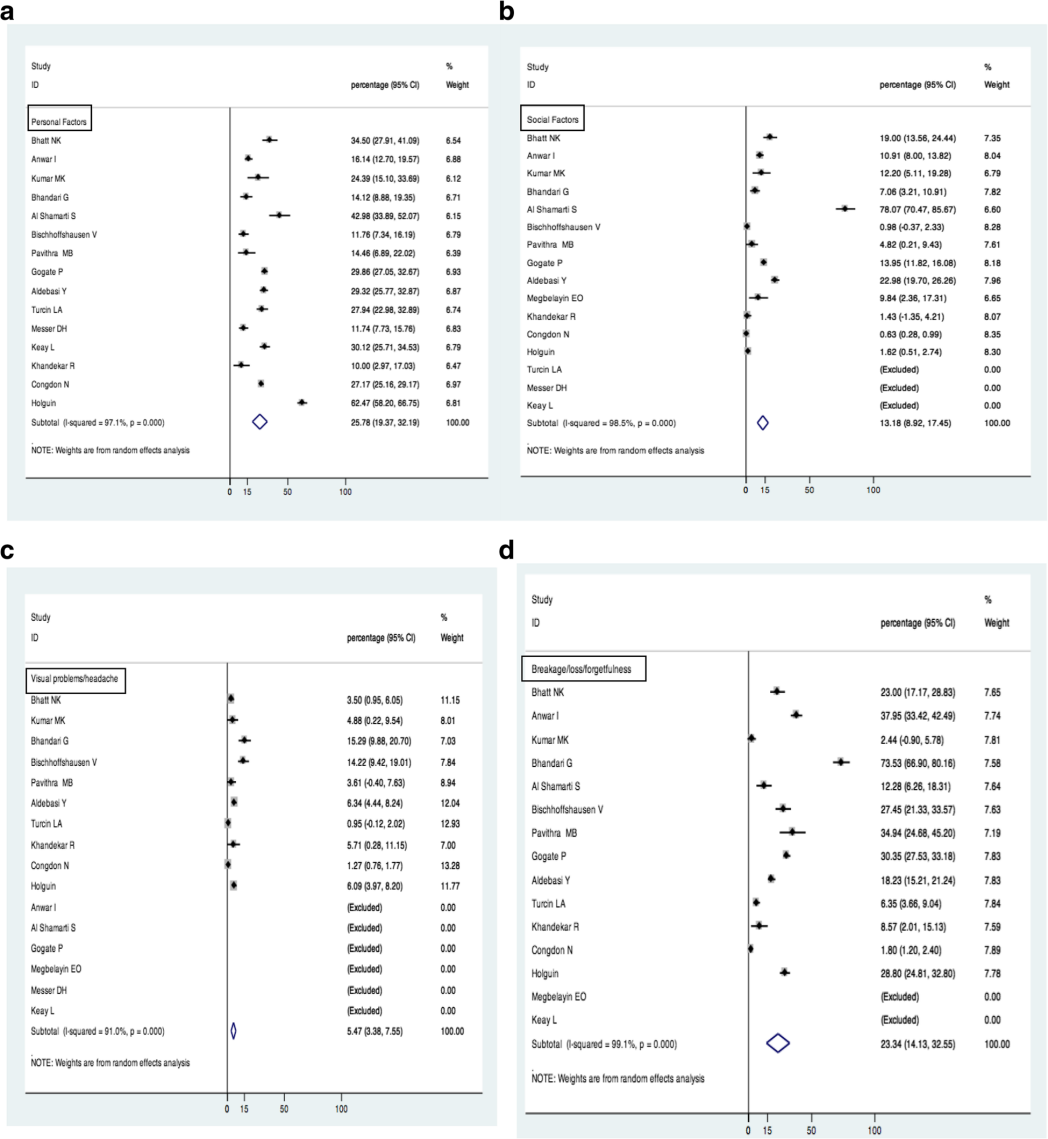
**a** Personal factors as a reason for non-compliance to spectacle use. **b** Social factors as a reason for non-compliance to spectacle use. **c** Visual problems/ headache as a reason for non-compliance to spectacle use. **d** Breakage/ loss/ forgetfulness as a reason for non-compliance to spectacle use

The most commonly cited reasons for non-compliance with spectacle use in the studies were broken [9–12, 14, 15, 17–20, 23, 24, 27]/lost spectacles [9–12, 14, 15, 17–20, 24, 27], forgetfulness [9–11, 14, 15, 17–19, 24, 27] and parental disapproval [8, 9, 11, 12, 15, 16, 18, 23, 24, 34]. These were followed by headache [10–12, 15, 17, 23, 24, 27], teasing by peers [10, 12, 19]and dislike for spectacles [9, 14, 16–23]. Other reasons mentioned in a few studies were use only when required [10, 12] unclear vision [11, 12, 22, 27, 34], unattractive frames/poor appearance [8, 34], fear of injuries [8, 34], lack of affordability [9, 12, 16], uncomfortable spectacles [18, 34] and negative attitude of the society [8, 15, 21](eFig.4). The reasons for non-compliance were pooled for different studies, and is as shown in the forest plots (Fig. 4(a-d). The reasons were broadly classified into personal factors, social factors, visual problems and breakage/loss/forgetfulness. The non-compliance due to personal factors was25.78% (Fig. 4a), for social factors it was 13.18% (Fig. 4b), visual problems/headache it was 5.47% (Fig. 4c) and for breakage/loss/forgetfulness it was 23.34% (Fig. 4d) of the total. The results clearly show that personal factors and breakage/loss/forgetfulness were the most commonly cited reasons for noncompliance.

## Discussion

The overall pooled estimate from twenty studies shows that the compliance with spectacle use is considerably low among children 40.14% (95% CI- 32.78-47.50), though the studies were heterogeneous. Majority of the studies were from South East Asia Region (SEAR) and lower middle-income countries. There was a dearth of studies from low-income countries, which could have altered the results and improved representativeness. The estimated compliance rate with spectacle was less than half (40.14%). Sub optimal compliance is a point of concern and can lead to progression of refractive errors. Negative, non significant correlation of percentage compliance with per capita GDP and percentage expenditure on health, suggests that apart from economic factors, psychosocial factors may be a contributor to compliance. It was observed that the setting (screening vs clinical care) and the method of assessing compliance (interview vs observation) did not have significant effect on the compliance. Although, pooled compliance in the clinical care setting where problem was identified by the child or family compared to vision screening setting provided at school was not significant, but the results point to of better compliance in clinical care setting, thus indicating that they are more likely to be aware and are motivated to use the spectacles. Personal, behavioral and cultural factors influence children’s compliance with spectacle use. The rate was less than half for most countries except a few which showed extremes on the lower and higher side. Compliance rate was overlapping between almost all studies except for two studies which reported a very high compliance [23, 25] and two that reported low compliance [16, 24]. In the study by Khandekar*et al* [23], the compliance may be high for two reasons. First, the sample size was small (77) that may have been a contributor to the difference in the compliance value from other studies. Second, the cut off for spectacle compliance was > 0.75D error, hence free glasses were given to children with higher refractive error, which itself is a factor for good compliance. In the study by Khandekar *et al* [25], the students were being assessed for compliance at regular intervals, thus improving the compliance rate. On the other hand, the reasons reported by Megbeylian *et al* [16] (2013) for poor compliance were lack of affordability and deep-rooted customs /traditions. In Nigeria, the expenditure on health as percentage of GDP and the HDI are low, which may be other contributory factors to affordability. The prescription cut offs for spectacle compliance assessment also varied across studies, though most studies did not report specific cut offs, a few showed that the compliance was poorer in children who had lower refractive errors as compared to children with higher refractive errors. Increased severity of refractive error warrants a stricter compliance to spectacle use as it hinders daily activities due to poor visibility. Some studies [14, 16, 19, 24, 27]reported better compliance in myopia as compared to hypermetropia. Recent study by Mc Cormick I et al. (2018) [35] also reported better compliance with higher refractive error in their study on determinants of compliance to spectacle use. Due to different definitions and cut offs we could not find an effect of these factors on the compliance as some studies have mentioned the prescription cut offs in terms of diopters while others have measured it in terms of visual acuity In addition the available data was also not uniform to be pooled together.

Identifying reasons for non-compliance with spectacle use is important for understanding the social determinants for intervention. The most commonly reported reasons for non-compliance were broken glasses [9–12, 14, 15, 17–20, 23, 24, 27], forgetfulness [9–11, 14, 15, 17–19, 24, 27], loss of spectacles [9–12, 14, 15, 17–20, 24, 27] and parental disapproval [8, 9, 11, 12, 15, 16, 18, 23, 24, 34].(Fig. 4 a- 4d) Addressing these issues by generating awareness is imperative.. The outcomes indicate more of socio cultural factors as major contributors to poor compliance that is commonly seen in Upper middle income countries. Any habit, if inculcated in the early years of life is bound to show results in adulthood. Most of the reasons identified for poor compliance are modifiable and are due to carelessness and poor encouragement of children. Breakage, loss and forgetfulness are intervention points that can bring substantial difference in the compliance rate. Parental disapproval is a significant contributor to child behavior. In many low and middle income countries, spectacles are considered a sign of weakness and their use hinders the process of finding a suitable match for the children when they reach adulthood. LASIK has been considered as a procedure for permanent removal of eyeglasses by surgical correction. Factors related to visual problems and headache can be addressed by modification of the prescription glasses and appropriate correction till comfort is achieved.

Considering the fact that low compliance with spectacle use in children could result in detrimental outcomes, this issue requires necessary and urgent action. Unless social and perceptual barriers are overcome, the families will not access the financial and logistical assistance available to seek eye care for school-aged children. Behavior change communication (BCC) targeted at education and behavior change of parents, so they encourage children to use spectacles, is advocated. Another point of action could be school health programs, which should focus on incorporating the component of ensuring compliance through follow-up, apart from screening of children for refractive errors. Some potential actions that are recommended for the poor compliance found in our studyinclude, provision of spectacles to children at zero cost, facilitation of school vision screening programs by government and involvement of teachers in identification of non compliant children. Parental education and support are key pillars to strengthen the interventions. It is unlikely that uni-dimensional intervention approaches to increase follow up and spectacle adherence in the context of refractive errors (e.g., free spectacles) will be adequate to achieve sustained improvement in treatment outcomes among children. Positive reinforcement is essential at both the school and household levels. Generating awareness and glamourizing spectacles by using lightweight, unbreakable and trendy frames will promote their acceptance, especially in adolescent age groups. Lastly, the role of eye care practitioners is imperative in early identification, diagnosis and treatment of refractive errors among children, so as to curb the problem at a very nascent stage.

There are a number of strengths of this study. First, the present review is a first systematic review on spectacle compliance in children. No systematic review or meta-analysis has been conducted previously on this topic. Efforts have been made to include all the available studies on the topic. Secondly, no time restriction was imposed and we have obtained studies for all years. There are a few limitations of the study as well. Despite all our efforts to extract maximum number of studies, we may have missed relevant studies in unpublished literature (publication bias). Also, the number of studies obtained was mostly from middle-income countries and a clearer picture of influential factors from high income and low-income countries would not be made very clear.

## Conclusions

In conclusion, this systematic review and meta-analysis has thrown light upon compliance with spectacle use and reasons poor compliance with spectacles which are a low cost intervention for refractive errors management in children. This issue needs to be addressed through behavioral motivation of children, parents and the society. This review can help stakeholders and program managers in defining health care interventions to improve compliance with spectacle use.

## Supplementary information

**Additional file 1: eTable 1**: Quality Assessment tool (modified for the study). **eTable 2:** Quality assessment of studies selected for the review. **eFig.1** Forest plot showing the effect of setting (screening vs clinical care) on the compliance with spectacle use. **eFig.2** Forest plot showing the effect of assessment method (interview vs observation) on the compliance with spectacle use. **eFig.3** Galbraith plot for statistical heterogeneity amongst studies. **eFig 4.** Reasons for Non-compliance to spectacle use.

## Data Availability

The datasets used and/or analysed during the current study are available from the corresponding author on reasonable request.

## Abbreviations

BCC: Behavior Change Communication
CI: Confidence Interval
GDP: Gross Domestic Product
HDI: Human Development Index
HIC: High Income Country
LIC: Low Income Country
LMIC: Lower Middle Income Country
MOOSE: Meta- analysis of Observational Studies in Epidemiology
PRISMA: Preferred Reporting Items for Systematic Reviews and Meta-analyses
RE: Refractive Error
SEAR: South East Asia Region
UMIC: Upper Middle Income Country
