# Supplementary material for: Compliance to spectacle use in children with refractive errors- a systematic review and meta-analysis
Source: BMC Ophthalmol. 2020 Feb 24;20:71. doi: 10.1186/s12886-020-01345-9 (PMC7038539; doi:10.1186/s12886-020-01345-9)
Supplement: Supplementary file 1 — Additional file 1: eTable 1: Quality Assessment tool (modified for the study). eTable 2: Quality assessment of studies selected for the review. eFig.1 Forest plot showing the effect of setting (screening vs clinical care) on the compliance with spectacle use. eFig.2 Forest plot showing the effect of assessment method (interview vs observation) on the compliance with spectacle use. eFig.3 Galbraith plot for statistical heterogeneity amongst studies. eFig 4. Reasons for Non-compliance to spectacle use. [file 12886_2020_1345_MOESM1_ESM.docx]

**eTable 1**: Quality Assessment tool (modified for the study)

| S.No. | Question | Description | Score |
| --- | --- | --- | --- |
| 1. | Was the sampling method representative of the population intended to the study | A. Non probability sampling (including: purposive, quota, convenience and snowball sampling)  B. Probability sampling (including: simple random, systematic, stratified, cluster, two-stage and multi-stage sampling) | 0  1 |
| 2. | How was non-response addressed? | A. Reasons for non- response described  B. Reason for non-response not described | 1  0 |
| 3. | Did the study report any response rate? (If the reported response rate is less than 60%, the question should be answered ‘No’.) | A. Yes  B. No | 1  0 |
| 4. | Was the measurement tool used valid and reliable? | A. Yes  B. No | 1  0 |
| 5. | What was the source of the data? | A. Secondary source: survey not specially designed for the purpose  B. Primary source | 0  1 |
| 6. | Do the authors include the definition of compliance used for their study? | A. Yes  B. No | 1  0 |
| 7. | Is Compliance further explored in the study? | A. Yes  B. No | 1  0 |

Scoring method: total score divided by total number of all applicable items.

Grading of the quality assessment checklist: 0–33% 33– 66% 67–100%

Poor Satisfactory Good

**eTable 2:** Quality assessment of studies selected for the review.

| S.No. | Authors | Q1 | Q2 | | Q3 | Q4 | | Q5 | Q6 | Q7 | | Score | Grade |
| --- | --- | --- | --- | --- | --- | --- | --- | --- | --- | --- | --- | --- | --- |
| 1. 1. | Narayanan A (May 2017) | 0 | 0 | | 1 | 1 | | 1 | 0 | 1 | | 57 | Satisfactory |
|  | Kumar MR (Apr 2017) | 0 | 0 | | 0 | 0 | | 1 | 0 | 1 | | 28.5 | Poor |
|  | Bhatt NK et al (Mar 2017) | 0 | 0 | | 0 | 0 | | 1 | 0 | 1 | | 28.5 | Poor |
| 1. 1. | Anwar I et al (Jan 2017) | 1 | 0 | | 0 | 0 | | 1 | 1 | 1 | | 57 | Satisfactory |
|  | Bhandari G et al (Dec 2016) | 1 | 1 | | 1 | 0 | | 1 | 1 | 1 | | 85.7 | Good |
| 1. 2. | Sumana M (Nov 2015) | 1 | 0 | | 1 | 0 | | 1 | 0 | 0 | | 42 | Satisfactory |
|  | Al Shamarti SA (Apr 2015) | 0 | 0 | 0 | | | 0 | 1 | 0 | | 1 | 28.5 | Poor |
| 1. 5. | von-Bischhoffshausen Et al(Oct 2014) | 1 | 1 | 1 | | | 0 | 1 | 1 | | 1 | 85.7 | Good |
|  | Pavithra MB et al (Apr-June 2014) | 0 | 0 | 1 | | | 0 | 1 | 0 | | 1 | 42 | Satisfactory |
|  | Aldebasi YH (Nov 2013) | 0 | 0 | 1 | | | 0 | 1 | 1 | | 1 | 57 | Satisfactory |
|  | Megbelayin EO (May 2013) | 1 | 1 | 1 | | | 0 | 1 | 0 | | 1 | 71.4 | Good |
|  | Turcin LA et al (Feb 2013) | 0 | 0 | 0 | | | 0 | 1 | 0 | | 1 | 28.5 | Poor |
| 1. 6. | Gogate P (Jan 2013) | 1 | 1 | 1 | | | 0 | 1 | 0 | | 1 | 71.4 | Good |
| 1. 7. | Messer DH et al (Jan 2012) | 1 | 1 | 1 | | | 0 | 1 | 0 | | 1 | 71.4 | Good |
| 1. 11. | Keay L et al (Oct 2010) | 1 | 0 | 1 | | | 0 | 1 | 1 | | 1 | 71.4 | Good |
|  | Li L (June 2010) | 1 | 0 | 1 | | | 1 | 1 | 0 | | 1 | 71.4 | Good |
| 1. 12. | Congdon N (Dec 2008) | 1 | 1 | 1 | | | 0 | 1 | 0 | | 1 | 71.4 | Good |
| 1. 13. | Liping Li et al (July 2008) | 1 | 1 | 1 | | | 0 | 1 | 1 | | 1 | 85.7 | Good |
|  | Khandekar R (2008) | 1 | 0 | 0 | | | 0 | 1 | 0 | | 1 | 42 | Satisfactory |
| 1. 14. | Odedra N et al (Sept 2007) | 1 | 0 | 0 | | | 0 | 1 | 0 | | 1 | 42 | Satisfactory |
| 1. 15 | Holguin AMC et al (March 2006) | 0 | 1 | 1 | | | 0 | 1 | 0 | | 1 | 57 | Satisfactory |
| 1. 16. | Khandekar R et al (2002) | 0 | 1 | 1 | | | 0 | 1 | 0 | | 1 | 57 | Satisfactory |
| 1. 18. | Horwood AM (1998) | 1 | 1 | 1 | | | 0 | 1 | 0 | | 1 | 71.4 | Good |

**eFig.1** Forest plot showing the effect of setting (screening vs clinical care) on the compliance with spectacle use


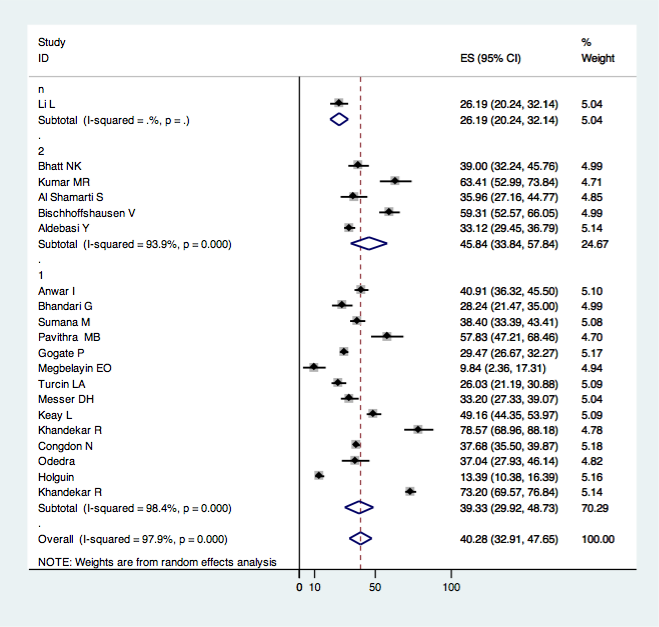


n= not applicable

1= clinical care

2= screening

**eFig.2** Forest plot showing the effect of assessment method (interview vs observation) on the compliance with spectacle use


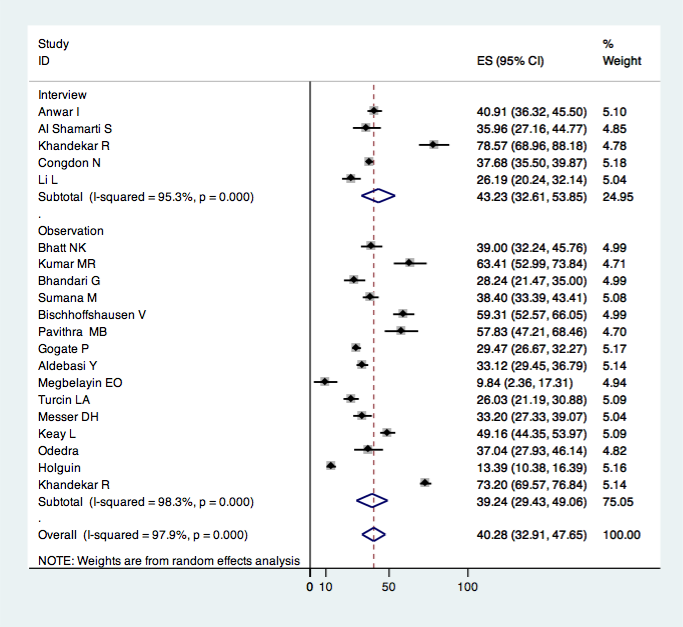


**eFig.3** Galbraith plot for statistical heterogeneity amongst studies


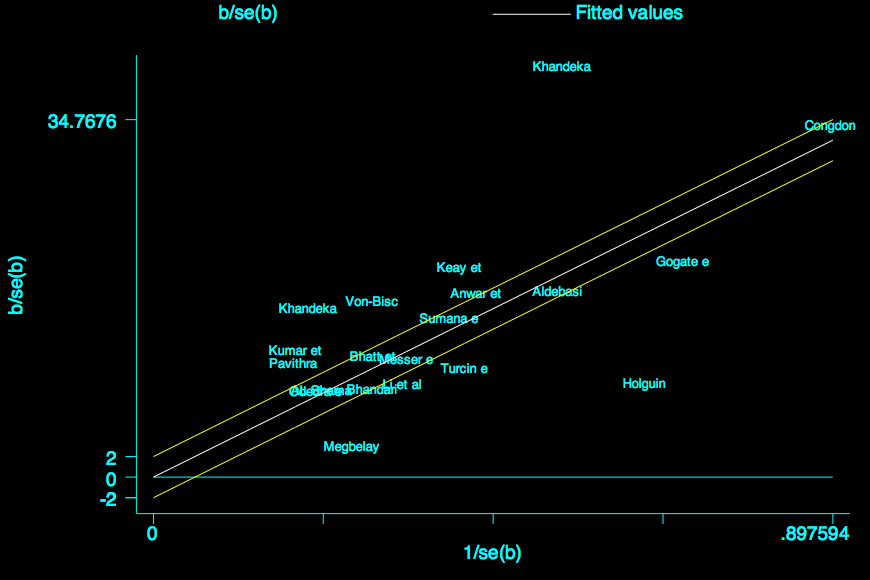


**eFig 4.** Reasons for Non-compliance to spectacle use
